# Effects of tea infusion on selenium uptake in grapevine

**DOI:** 10.1186/s12870-024-05379-9

**Published:** 2024-07-10

**Authors:** Jin Wang, Yunying Xiao, Dilian Zhang, Zhen Dai, Kewen Huang, Xun Wang, Xiulan Lv, Lijin Lin

**Affiliations:** 1 College of Horticulture, Sichuan Agricultural University, Chengdu, 611130 China; 2 Institute of Horticulture, Chengdu Academy of Agriculture and Forestry Sciences, Chengdu, 611130 China

**Keywords:** Fruit tree, Physiology, Element absorption, Water extract

## Abstract

Increased selenium (Se) content in fruits can supply Se in human body, but the effects of teas on the Se uptake in fruit trees are unknown. The effects of infusions of four teas (green, black, dark, and white) on the Se uptake of grapevine were studied to promote the Se uptake in fruit trees in this study. However, only black tea infusion increased the biomass, photosynthetic pigment content, superoxide dismutase (SOD) activity, peroxidase (POD) activity, and soluble protein content of grapevine. Except for white tea infusion, other tea infusions also increased the catalase (CAT) activity of grapevine. Furthermore, the tea infusions increased the activities of adenosine triphosphate sulfurase (ATPS) and adenosine 5’-phosphosulfate reductase (APR), and decreased the activities of serine acetyltransferase (SAT) and selenocysteine methyltransferase (SMT). Only the dark and white tea infusions increased the shoot total Se content by 86.53% and 23.32%, respectively (compared with the control), and also increased the shoot inorganic Se content and shoot organic Se content. Notably, four tea infusions decreased the organic Se proportion and increased the inorganic Se proportion in grapevine. Correlation and grey relational analyses showed that the root total Se content, ATPS activity, and ARP activity were closely associated with the shoot total Se content. The principal component and cluster analyses also showed that the ATPS activity, APR activity, root total Se content, and shoot total Se content were classified into one category. These findings show that black tea infusion can promote grapevine growth, while dark and white tea infusions can promote the Se uptake in grapevine.

## Introduction

Selenium (Se) is important for human body, and Se deficiency is less than 40 µg/d and Se poisoning is more than 400 µg/d [1–3]. Se mainly exists in inorganic forms and biomolecules bound to organic Se. Compared with inorganic Se, organic Se is safer, biologically active, and more efficient [4]. Se (organic Se) is mainly obtained through vegetables and fruits, the Se-enriched horticultural crops can supply the required Se for human body [5, 6]. So, the increase of Se content in horticultural crops can prevent the Se deficiency of human body. There are various methods that can be used to increase the Se content in horticultural crops, and Se fertilizer is the most widely used method, but the long-term use of Se fertilizer can become toxic for horticultural crops [7]. Therefore, screening of some agronomic measures is necessary for improving the Se levels in vegetables and fruits [8].

Tea [*Camellia sinensis* (L.) O. Kuntze] is classified as green, white, yellow, oolong, black, and dark according to the manufacturing methods and flavor of products [9]. Among these tea types, green tea is an unfermented tea, white tea is a lightly fermented tea, black tea is a fully fermented tea, and dark tea is a post-fermented tea [10, 11]. Tea contains active organic substances, including polyphenols, polysaccharides, amino acids, alkaloids, and vitamins [12]. Tea polyphenols have antioxidant effects, and can inhibit microbial activity, and can be used for soil improvement [13, 14] by reducing the activity of toxic metals [15] and activating soil mineral elements [16]. The biochar of tea residues can increase soil organic carbon content, oxidative stability of soil organic carbon, and soil water-stable agglomerate stability [17, 18]. Tea residues also can promote the growth and quality of baby mustard [19], and promote the growth and yield of peppers and tomatoes [20, 21]. The polyphenols and caffeine in teas can reduce the aluminum toxicity on grapes [22]. Tea application can decrease cadmium absorption and enhance the resistance of pakchoi cabbage in cadmium-contaminated soil [23]. However, the effects of teas on the Se uptake in horticultural crops are unknown.

Grape (*Vitis vinifera* L.) has great economic and nutritional value, and is widely used for fresh food and processing [24]. However, grapes have low Se enrichment capacity [25]. Improving the Se content in grapes can improve their commodity value [26]. If application of tea infusion on grapes, the Se enrichment capacity in grapes could be improved, but there are no reports about that. So, we applied the infusions of four teas (black, green, white, and dark) on grape seedlings, and the effects of tea infusion on the Se uptake of grapevine were studied. This study aimed to screen the suitable tea infusion that could increase the Se content in grapes, and take a reference for Se-rich grape production.

## Materials and methods

### Materials

The branches of ‘Summer Black’ grape were cut in December 2021 and stored in sands. The branches were cut into 15 cm in length in February 2022, and the plug trays with perlite were planted in a greenhouse with the conditions of Li et al. [27] until the new shoots grew to 15 cm.

Fluvo-aquic soil samples were collected from the farmland of Sichuan Agricultural University (30°42′N, 103°51′E). The basic physicochemical properties of fluvo-aquic soil were previously described, and the soil Se content was 0.35 mg/kg [28].

Green, black, dark, and white teas were used in this experiment, and obtained the same as Zhang et al. [29]. All these tea types were produced by *C. sinensis*. The tea infusions were made according to the method of Zhang et al. [29], and the Se in tea infusion was not detected.

### Experimental design

The experiment was conducted in a canopy. The soil samples were treated (February 2022) and mixed as described by Lin et al. [30], then, the Se (Na_2_SeO_3_) was added into the soil at the final soil Se concentration of 5 mg/kg and mixed [28, 31]. Three uniform grape seedlings were planted into each pot, and tea infusion (30 mL) was irrigated into each pot after ten days of planting. A total of five treatments were conducted: no tea infusion (control), green tea infusion, black tea infusion, dark tea infusion, and white tea infusion. Each treatment had three repeats (two pots as a repeat). The same volume of tea infusion was irrigated every seven days (four times in total).

### Determination of indicators

One month after the first application of tea infusion, one mature leaf of grapevine was collected for further analysis. The contents of photosynthetic pigments (chlorophyll *a*, chlorophyll *b*, total chlorophyll, and carotenoid) were measured via acetone-ethanol extraction method. The activities of antioxidant enzymes, including superoxide dismutase (SOD), catalase (CAT), and peroxidase (POD) were detected via the nitrotetrazole chloride reduction, potassium permanganate titration, and guaiacol methods, respectively. Soluble protein content was measured via the Coomassie brilliant blue method as described by Hao et al. [32]. The activities of Se metabolism-related enzymes, including adenosine triphosphate sulfurase (ATPS), adenosine 5’-phosphosulfate reductase (APR), serine acetyltransferase (SAT) and selenocysteine methyltransferase (SMT), were determined using the enzyme-linked immunosorbent assay (ELISA) kits (Shanghai Enzyme Link Biotechnology Co., Ltd., Shanghai, China) following the manufacturer’s instructions. After that, the whole grape seedlings were removed, cleaned, and dried as described by Li et al. [27]. The root and shoot dry weights (biomass) were measured using an electronic balance. The dried samples were ground for determining the total Se and inorganic Se contents. The finely ground samples (1.000 g) were digested with nitrate acid: perchloric acid (4:1, v/v) and reduced with hydrochloric acid, and the digested solutions were used to determine the content of Se using hydride generation-atomic fluorescence spectrometry (AFS-9700; Beijing Haiguang Instrument Co., Ltd., Beijing, China) as described by Li et al. [27]. Another finely ground samples were extracted with 6 mol/L hydrochloric acid, and the extracted solution was used to determine the content of inorganic Se using hydride generation-atomic fluorescence spectrometry according to Li et al. [27]. The measured values of Se were checked by using certified standard reference material (GBW-07602, bush branches and leaves) obtained from the China National Center for Standard Reference Materials. The organic Se content was calculated as follows: organic Se content = total Se content - inorganic Se content [27]. The translocation factor (TF) was calculated as follows: shoot Se content/root Se content [33].

### Statistical analysis

Statistical analyses were conducted using software SPSS 20.0.0 (IBM, Chicago, IL, USA). Data were normalized and subjected to a homogeneity test before one way analysis of variance and Duncan’s multiple range test (*P <* 0.05). The correlations among all indicators were assessed using Pearson’s correlation analysis. The grey relational analysis was used to analyze the grey correlations of the different indicators with the shoot total Se content, as described by Lin et al. [34] and Zhang et al. [35]. Data of each indicator were subjected to the principal component analysis (PCA) and cluster analysis as described by Lin et al. [34].

## Results

### Biomass (dry weight) of grapevine

**Fig. 1:**
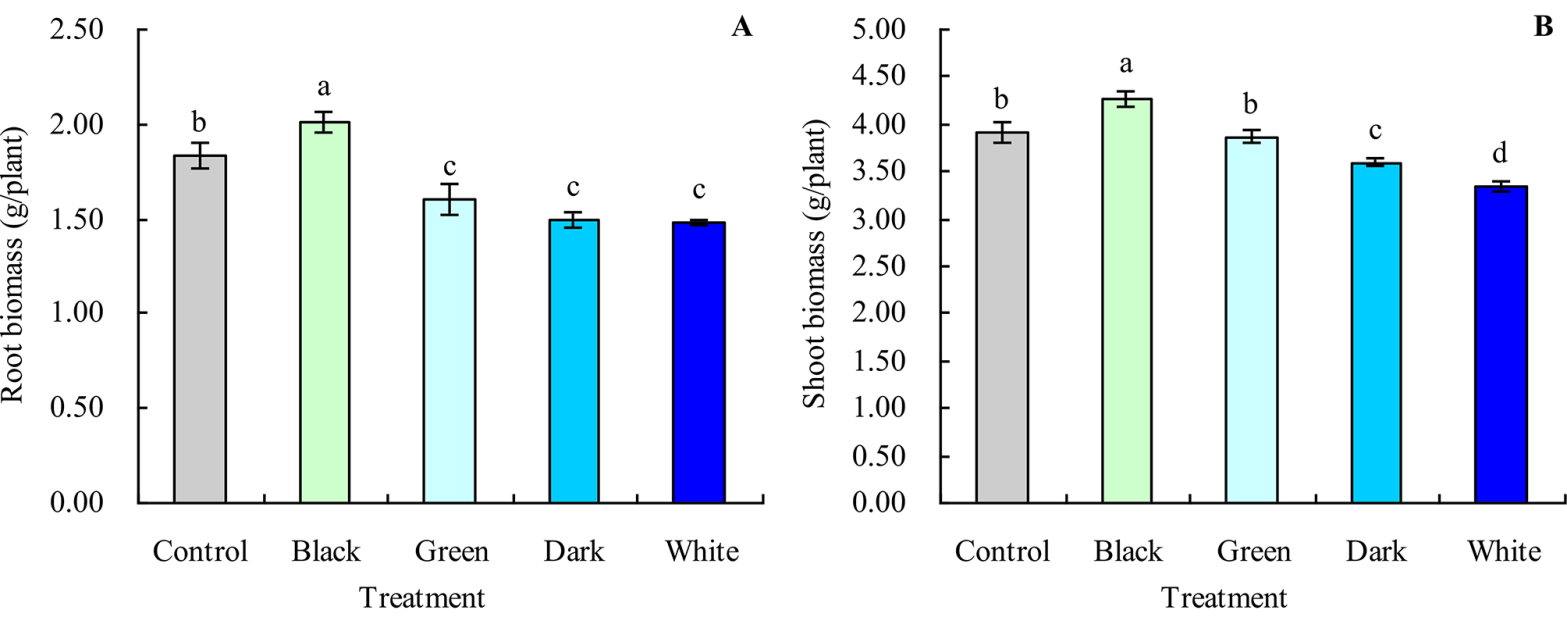
Biomass (dry weight) of grapevine. **A**: root biomass; **B**: shoot biomass. Values are means ± SE of three biological replicates. Different lowercase letters indicate significant differences among the treatments (Duncan’s Multiple Range Test, *P* < 0.05)

Black tea infusion increased the root and shoot biomass of grapevine (Fig. 1A and B). Compared with the control, black tea infusion increased the root and shoot biomass by 9.46% and 8.76%, respectively, and green, dark, and white tea infusions decreased the root biomass by 12.65%, 18.75%, and 19.44%, respectively. Although green tea infusion had no significant effect on the shoot biomass, dark and white tea infusions decreased the shoot biomass.

### Photosynthetic pigment content in grapevine leaves

**Fig. 2:**
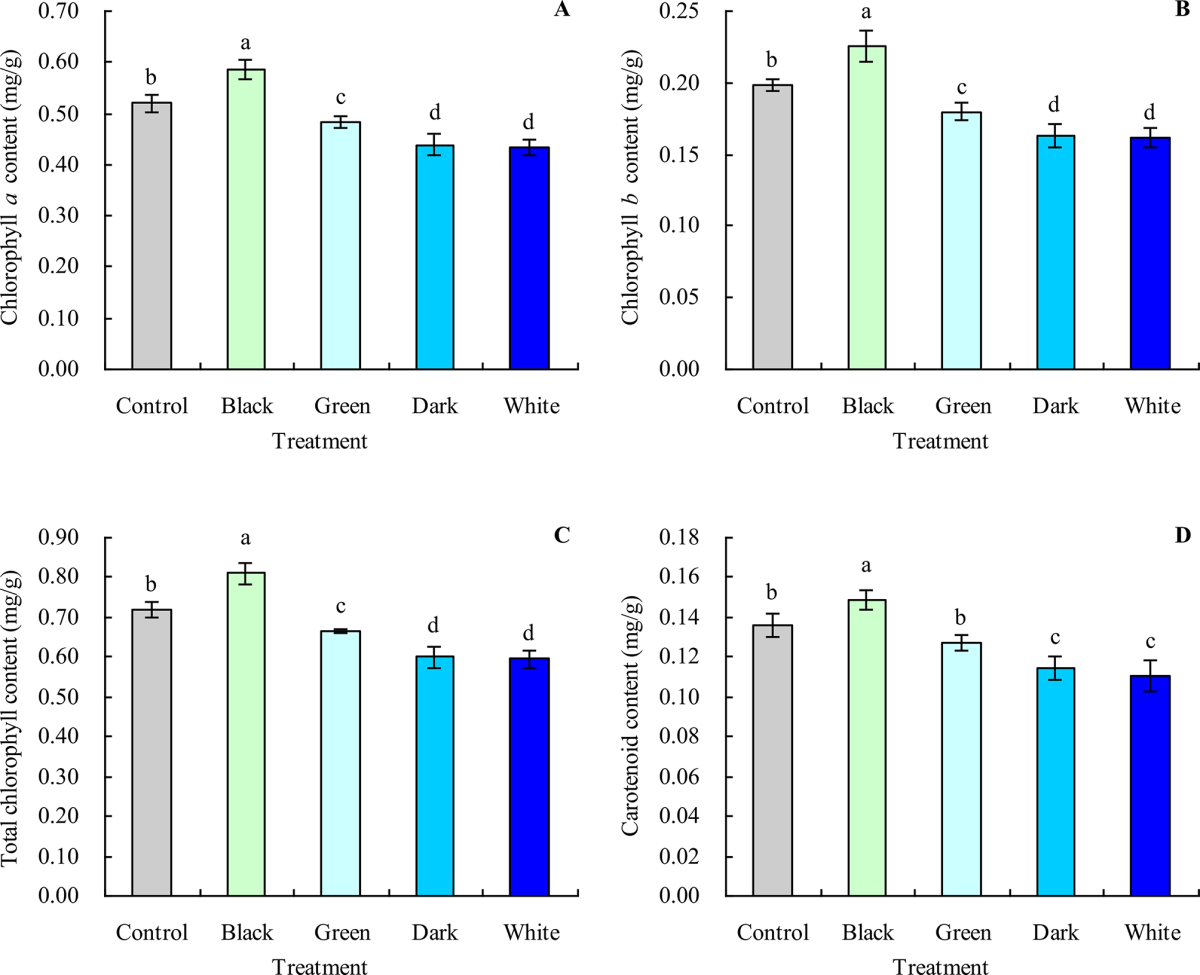
Photosynthetic pigment content in grapevine leaves. **A**: chlorophyll *a* content; **B**: chlorophyll *b* content; **C**: total chlorophyll content; **D**: carotenoid content. Values are means ± SE of three biological replicates. Different lowercase letters indicate significant differences among the treatments (Duncan’s Multiple Range Test, *P* < 0.05)

Compared with the control, black tea infusion increased the contents of chlorophyll *a*, chlorophyll *b*, total chlorophyll, and carotenoid in grapevine leaves by 12.50%, 13.64%, 12.81%, and 9.56%, respectively (Fig. 2A and D). In contrast, green, dark, and white tea infusions decreased the contents of chlorophyll *a*, chlorophyll *b*, and total chlorophyll. Although green tea infusion had no significant effect on the carotenoid content, dark and white tea infusions decreased the carotenoid content.

### Antioxidant enzyme activities and soluble protein contents of grapevine leaves

**Fig. 3:**
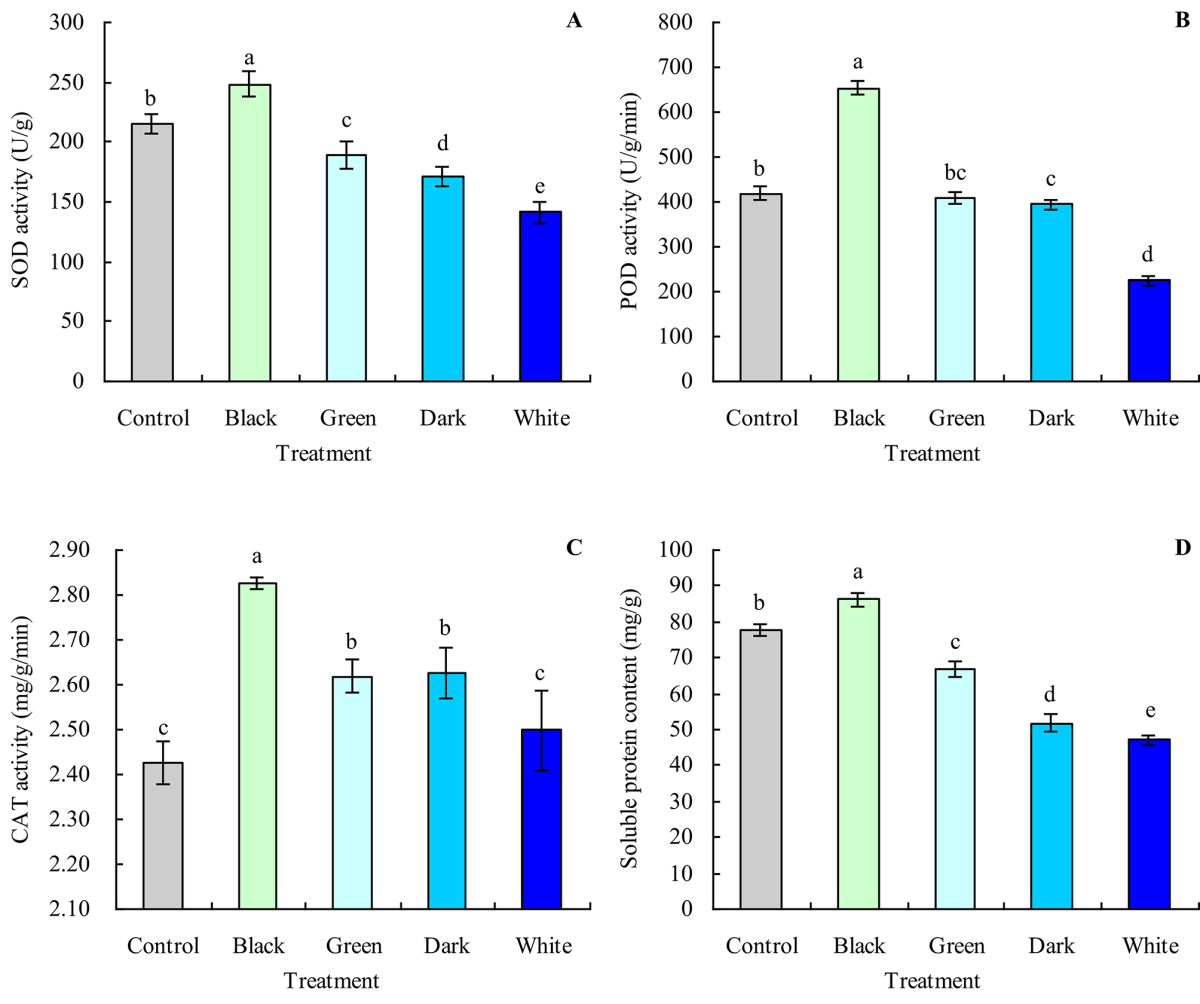
Antioxidant enzyme activities and soluble protein contents of grapevine leaves. **A**: SOD activity; **B**: POD activity; **C**: CAT activity; **D**: soluble protein content. Values are means ± SE of three biological replicates. Different lowercase letters indicate significant differences among the treatments (Duncan’s Multiple Range Test, *P* < 0.05)

Black tea infusion increased the SOD activity, POD activity, and soluble protein content of grapevine leaves by 15.56%, 55.87%, and 10.94%, respectively, compared with the control (Fig. 3A, B and D). In contrast, green, dark, and white tea infusions either decreased or had no significant effect on the SOD activity, POD activity, and soluble protein content. Black, green, and dark tea infusions increased the CAT activity by 16.45%, 7.96%, and 8.24%, respectively, compared with the control, while white tea infusion did not significantly affect the CAT activity (Fig. 3C).

### Se metabolism-related enzyme activity of grapevine leaves

**Fig. 4:**
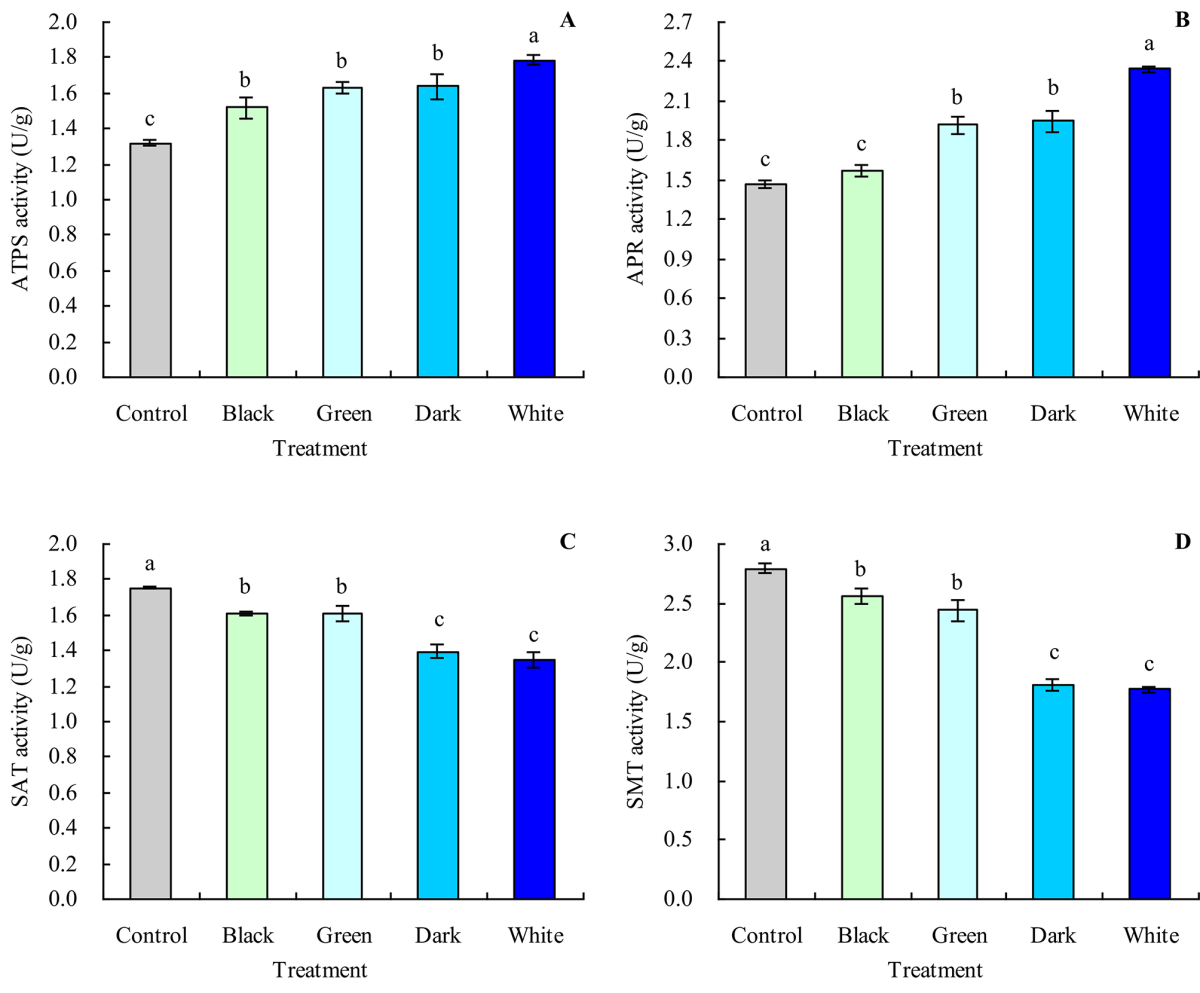
Se metabolism-related enzyme activity of grapevine leaves. **A**: ATPS activity; **B**: APR activity; **C**: SAT activity; **D**: SMT activity. Values are means ± SE of three biological replicates. Different lowercase letters indicate significant differences among the treatments (Duncan’s Multiple Range Test, *P* < 0.05)

Black, green, dark, and white tea infusions increased the ATPS activity of grapevine leaves and decreased the SAT and SMT activities (Fig. 4A, C and D). Compared with the control, black, green, dark, and white tea infusions increased the ATPS activity by 14.92%, 23.64%, 23.94%, and 35.23%, respectively, and decreased the SAT activity by 8.38%, 8.15%, 20.52%, and 23.32%, respectively. Also, black, green, dark, and white tea infusions decreased the SMT activity by 8.12%, 12.67%, 35.11%, and 36.44%, respectively (compared with the control). Furthermore, green, dark, and white tea infusions increased the APR activity by 30.50%, 32.40%, and 59.50%, respectively (compared with the control), while black tea infusion had no significant effect on the APR activity (Fig. 4B).

### Se content and translocation factor of grapevine

**Fig. 5:**
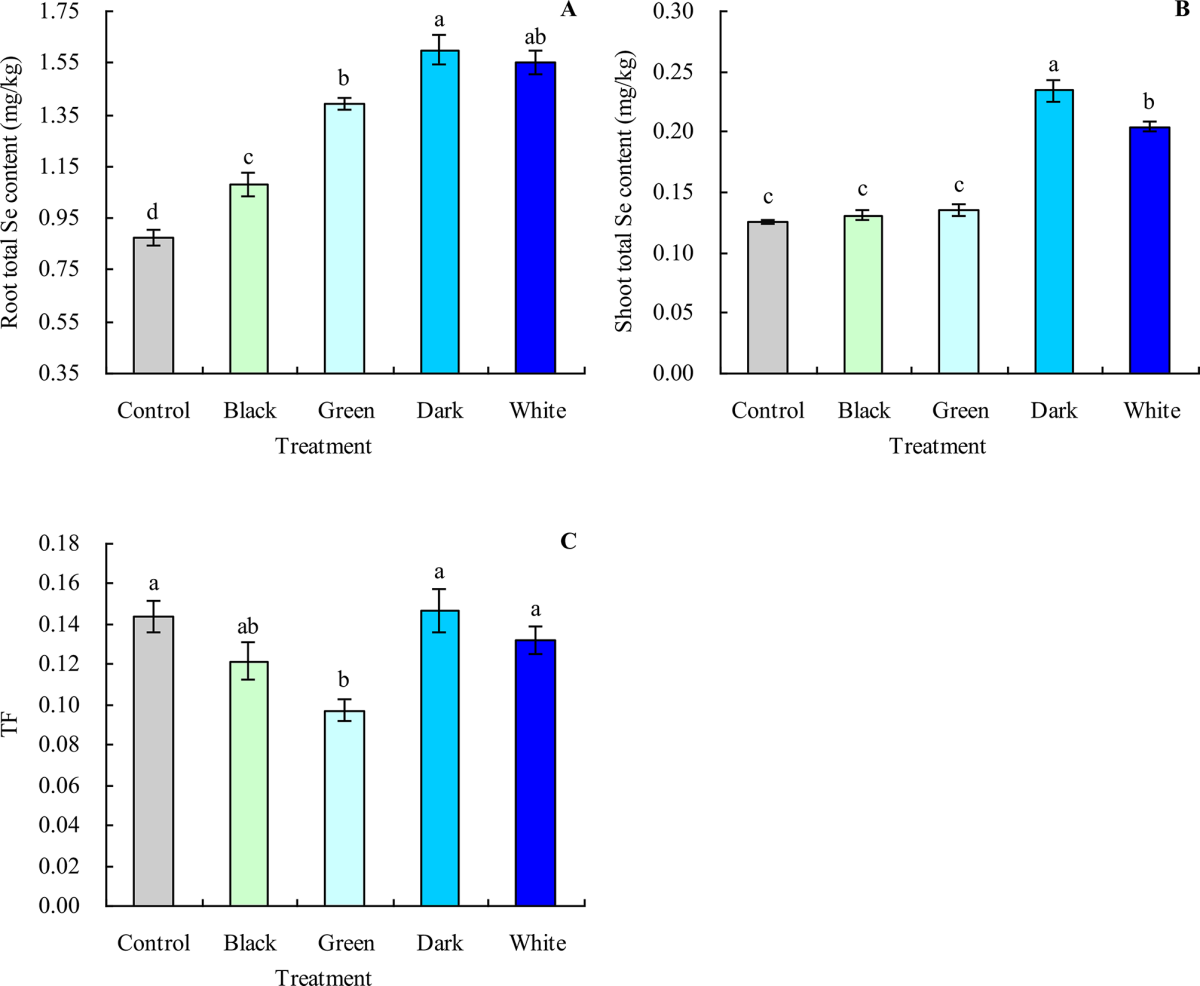
Total Se content and translocation factor of grapevine. **A**: root total Se content; **B**: shoot total Se content; **C**: translocation factor (TF). Values are means ± SE of three biological replicates. Different lowercase letters indicate significant differences among the treatments (Duncan’s Multiple Range Test, *P* < 0.05)

Black, green, dark, and white tea infusions increased the root total Se content in grapevine by 23.42%, 58.64%, 82.64%, and 77.00%, respectively, compared with the control (Fig. 5A). Although black and green tea infusions did not significantly affect the shoot total Se content, dark and white tea infusions increased the shoot total Se content by 86.53% and 23.32%, respectively (compared with the control) (Fig. 5B). Black and green tea infusions decreased the TF of grapevine, while dark and white tea infusions did not significantly affect the TF (Fig. 5C).

**Fig. 6:**
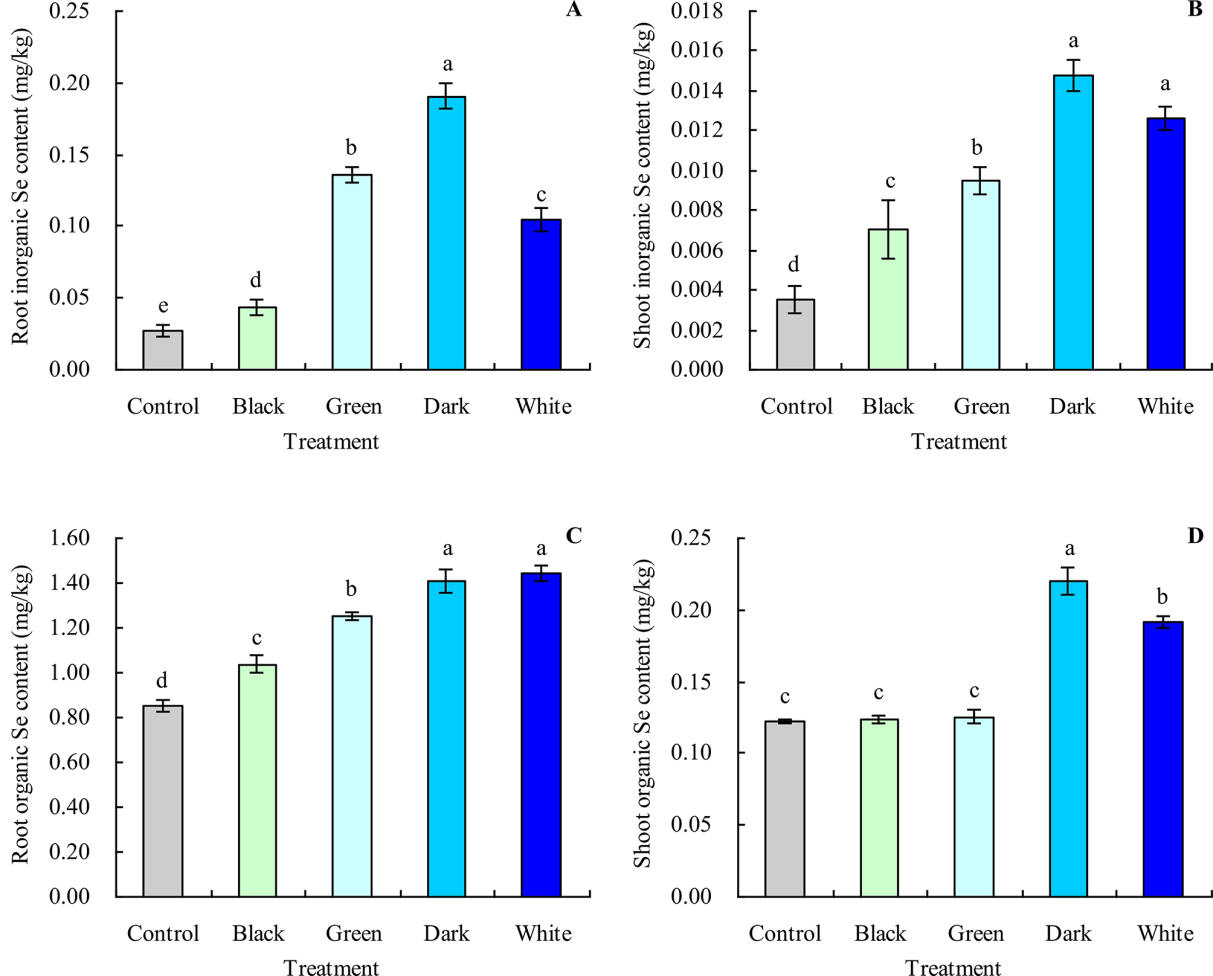
Inorganic Se and organic Se contents in grapevine. **A**: root inorganic Se content; **B**: shoot inorganic Se content; **C**: root organic Se content; **D**: shoot organic Se content. Values are means ± SE of three biological replicates. Different lowercase letters indicate significant differences among the treatments (Duncan’s Multiple Range Test, *P* < 0.05)

**Fig. 7:**
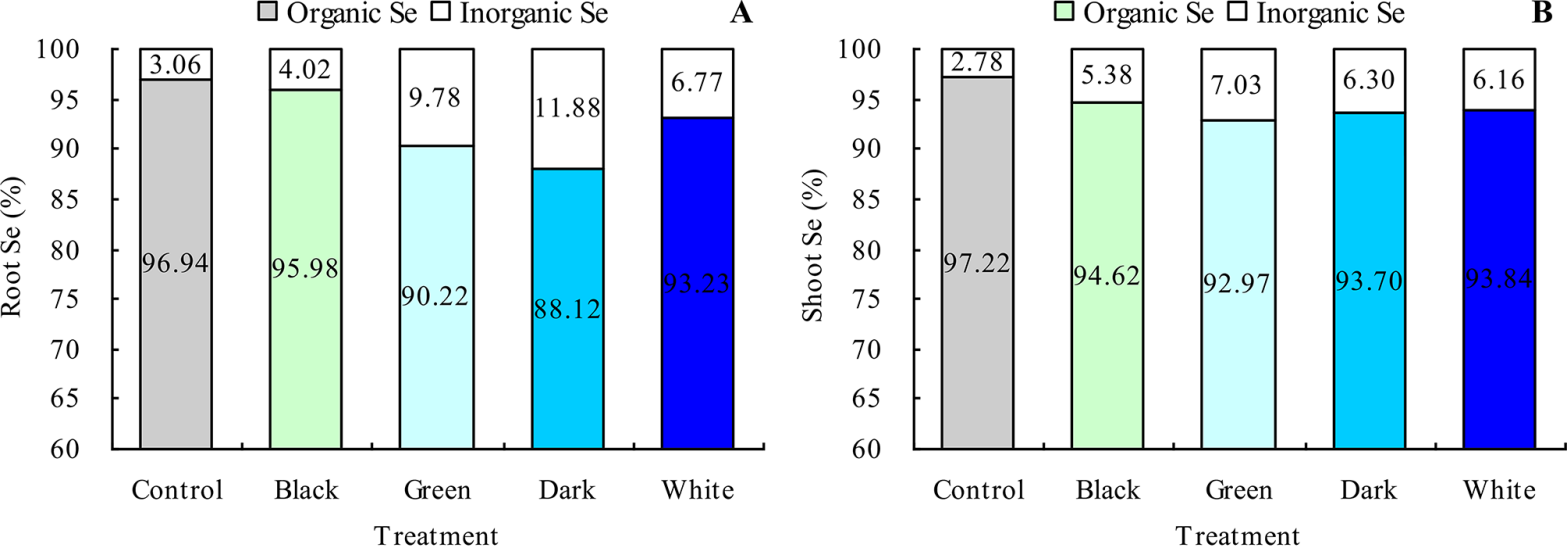
Proportions of organic Se and inorganic Se of grapevine. **A**: root Se proportion; **B**: shoot Se proportion

Black, green, dark, and white tea infusions increased the contents of root inorganic Se, shoot inorganic Se, and root organic Se in grapevine (Fig. 6A and C). Also, dark and white tea infusions increased the shoot organic Se content, while black and green tea infusions did not significantly affect the shoot organic Se content (Fig. 6D). The most Se form in grapevine was the organic Se (Fig. 7A and B). Black, green, dark, and white tea infusions decreased the root and shoot organic Se proportions and increased their inorganic Se proportions.

### Correlation and grey relational analyses

**Fig. 8:**
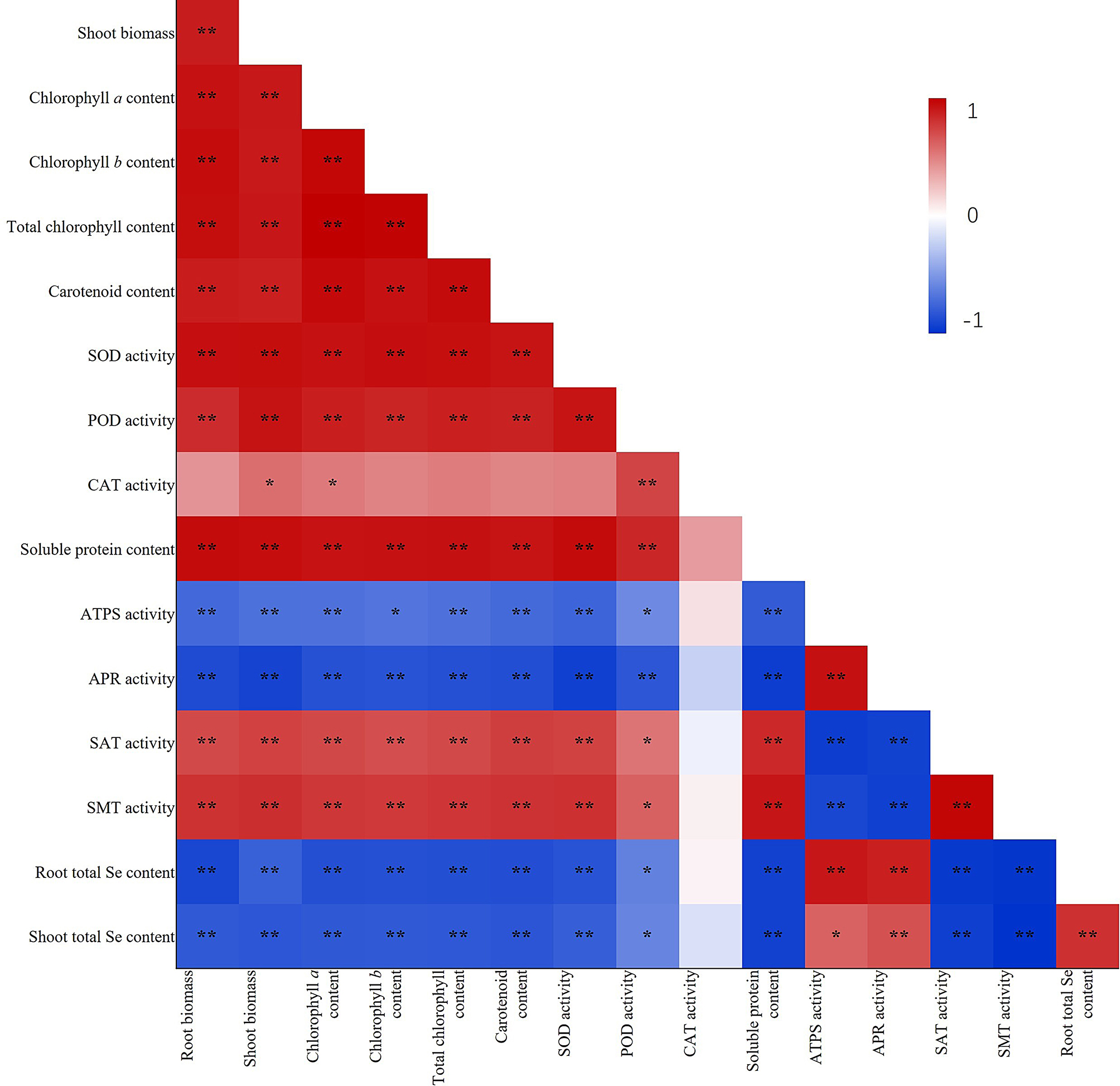
Correlations among the indicators. *N* = 15. **Correlation is significant at the 0.01 level (two-tailed test). *Correlation is significant at the 0.05 level (two-tailed test)

The root and shoot total Se contents were positively correlated with the activities of ATPS and APR (Fig. 8). However, the root and shoot total Se content were negatively correlated with the root biomass, shoot biomass, chlorophyll *a* content, chlorophyll *b* content, total chlorophyll content, carotenoid content, SOD activity, POD activity, soluble protein content, SAT activity, and SMT activity. Also, the root total Se content was positively correlated with the shoot total Se content.

**Fig. 9:**
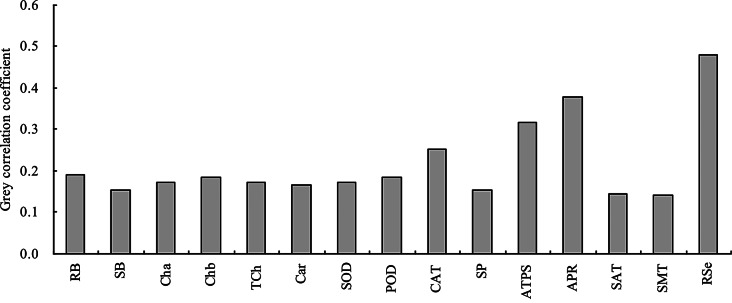
Grey correlation coefficient of the biomass, root total Se content, photosynthetic pigment content, antioxidant enzyme activity, soluble protein content, and Se metabolism-related enzyme activity with the shoot total Se content. RB = root biomass; SB = shoot biomass; Cha = chlorophyll *a* content; Chb = chlorophyll *b* content; TCh = total chlorophyll content; Car = carotenoid content; SOD = SOD activity; POD = POD activity; CAT = CAT activity; SP = soluble protein content; ATPS = ATPS activity; APR = APR activity; SAT = SAT activity; SMT = SMT activity; RSe = root total Se content

The grey relational analysis showed that the grey correlation coefficients of all indicators with the shoot total Se content were higher than 0.13 (Fig. 9), indicating that all indicators were correlated with the shoot total Se content. The root total Se content, ATPS activity, and ARP activity had the highest grey correlation coefficients, and were closely associated with the shoot total Se content.

### PCA and cluster analysis

**Fig. 10:**
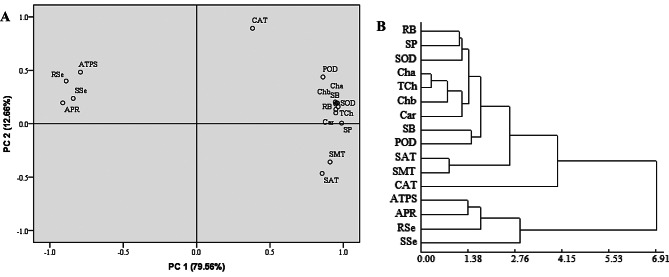
Principal component analysis (**A**) and cluster (**B**) analysis of each indicator. RB = root biomass; SB = shoot biomass; Cha = chlorophyll *a* content; Chb = chlorophyll *b* content; TCh = total chlorophyll content; Car = carotenoid content; SOD = SOD activity; POD = POD activity; CAT = CAT activity; SP = soluble protein content; ATPS = ATPS activity; APR = APR activity; SAT = SAT activity; SMT = SMT activity; RSe = root total Se content; SSe = shoot total Se content

To further study the classification of shoot total Se content with which indicators, PCA and cluster analysis were used in this study (Fig. 10A). PCA revealed that PC1 and PC2 accounted for 79.56% and 12.66% variance, respectively (total 92.22%). The ATPS activity, APR activity, root total Se content, and shoot total Se content were closely related. The SAT activity was closely related to SMT activity. Furthermore, The root biomass, shoot biomass, chlorophyll *a* content, chlorophyll *b* content, total chlorophyll content, carotenoid content, SOD activity, POD activity, and soluble protein content were closely related. However, the CAT activity was not closely associated with any indicator. In addition, cluster analysis grouped the indicators into four categories (Fig. 10B). The first cluster category comprised the root biomass, shoot biomass, chlorophyll *a* content, chlorophyll *b* content, total chlorophyll content, carotenoid content, SOD activity, POD activity, soluble protein content; the second comprised the SAT activity, and SMT activity; the third comprised the CAT activity; and the fourth comprised the ATPS activity, APR activity, root total Se content, and shoot total Se content. The PCA and cluster analysis had the same category.

## Discussion

Tea contains many active substances, minerals, and trace elements [36]. Tea polyphenols are the main antioxidant components in tea [37], which can increase the activity of enzymes in fruit trees [38, 39]. However, tea polyphenol contents at various fermentation degrees are different. Green tea has the highest high polyphenol content, while black tea has the lowest polyphenol content [10, 11]. Tea polyphenols can improve soil evolution and activation and migration of salt-based ions in paddy soils, thus improving soil nutrient effectiveness [14]. Tea can increase the yield and nutrient uptake of crops [40–42], and also can increase grape biomass, and increase grape and tomato chlorophyll contents [43, 44]. Although tea polyphenols can increase the biomass of grapes, high concentration negatively affects growth of grape rootstock and leaves [45]. In this experiment, only black tea infusion increased grapevine biomass and photosynthetic pigment contents. Green, dark, and white tea infusions slightly decreased grapevine biomass and photosynthetic pigment content. However, other previous studies showed different results [40–44], suggesting that different tea infusions have various effects on the growth of grapevine. The content of tea polyphenols is lower in black tea than in green, dark, and white teas [10, 11]. Furthermore, the contents of tea polyphenols in green, black, and white infusions may exceed the maximum tolerated by grapevine [45]. As a result, black tea infusion promoted grapevine growth, while the green, dark, and white tea infusions inhibited or did not significantly affect grapevine growth. Meanwhile, only black tea infusion increased the SOD activity, POD activity, and soluble protein content, while green, dark, and white tea infusions decreased or did not significantly affect these parameters. Also, black, green, and dark tea infusions increased the CAT activity, while white tea infusion did not significantly affect the CAT activity. Other previous studies also reported different results [38, 39], suggesting that black tea infusion can improve grapevine resistance to adversity. The differences in results may be related to the concentrations of tea polyphenols in tea infusions [10, 11, 45]. The mechanisms of different tea infusions on the growth and physiology of grapevine need further studied.

Plants mainly absorb Se in the inorganic Se forms, such as selenate and selenite [46, 47]. The inorganic Se is then converted into the organic Se after root absorption, with only a small content transferred to the aboveground parts, while most content remains in plant roots [48]. The levels of Se content in plants are related to absorption capacity, soil organic Se content, and soil pH value [49–51]. Tea can improve soil fertility and reduce the loss of mineral elements, such as nitrogen, phosphorus, potassium, and magnesium, from the soil [42]. Studies have also shown that tea polyphenols can improve the uptake of elements, such as manganese and calcium by plants [52, 53]. In this study, black, green, dark, and white tea infusions increased the ATPS and APR activities and decreased the SAT and SMT activities of grapevine. Furthermore, black, green, dark, and white tea infusions increased the different forms of root Se contents in grapevine. Also, dark and white tea infusions increased the different forms of shoot Se contents in grapevine. However, black and green tea infusions decreased the TF of grapevine, while dark and white tea infusions did not significantly affect the TF. These results suggest that tea infusion can promote Se accumulation in grapevine, especially dark and white tea infusions that can promote Se transporting to shoots of grapevine. The uptake ability may be because of the tea polyphenols content in different teas [10, 11], and tea infusions also change soil Se forms and soil pH value [49–51]. The contents of tea polyphenol in white and dark tea can change the Se metabolism-related enzyme activity, thus promoting Se transporting from roots to shoots of grapevine. Also, the high and low contents of tea polyphenol in green tea and black tea, respectively, have lower effects on Se uptake. Nonetheless, further studies are needed to evaluate the mechanisms by which tea infusions promote Se uptake in grapevine. Notably, results showed that Se mainly existed in the organic Se form in grapevine, consistent with previous studies [48]. Furthermore, tea infusions decreased the organic Se proportion in grapevine, and increased its inorganic Se proportion, thus can promote the transport of inorganic Se to above-ground part [48]. In addition, correlation analysis showed that the growth (biomass) and physiology indicators were negative correlated with the Se content, indicating that the Se uptakes in well-grown grapevines were lower than in weak grapevines. Correlation and grey relational analyses also showed that the root total Se content, ATPS activity, and ARP activity were closely associated with the shoot total Se content. PCA and cluster analysis also showed that ATPS activity, APR activity, root total Se content, and shoot total Se content were classified into one category, indicating that the root total Se content, ATPS activity, and ARP activity have the greatest effects on promoting Se uptake in aboveground parts of grapevine. Although the present study just investigated the Se accumulation in grapevine, the Se accumulation in berry fruits of grape could be also increased by dark and white tea infusions according previous studies [54, 55]. The promoting effects of the dark and white tea infusions on berry fruits of grape and their application time need to be further studied.

## Conclusion

Black tea infusion promoted grapevine growth by increasing the biomass, photosynthetic pigment content, antioxidant enzyme activity, and soluble protein content under Se-rich soils. Furthermore, dark and white tea infusions promoted grapevine Se uptake by increasing the different forms of shoot Se contents. Also, root total Se content, ATPS activity, and ARP activity are closely related to shoot total Se content. Nevertheless, the mechanism by which dark and white tea infusions increase the Se uptake in grapevine and the Se accumulation in berry fruits of grape requires further studies.

## Data Availability

No datasets were generated or analysed during the current study.
